# Palaeoecology and palaeophytogeography of the Rhynie chert plants: further evidence from integrated analysis of *in situ* and dispersed spores

**DOI:** 10.1098/rstb.2016.0491

**Published:** 2017-12-18

**Authors:** Charles H. Wellman

**Affiliations:** Department of Animal and Plant Sciences, University of Sheffield, Alfred Denny Building, Western Bank, Sheffield S10 2TN, UK

**Keywords:** Lower Devonian, Pragian, early land plants

## Abstract

The remarkably preserved Rhynie chert plants remain pivotal to our understanding of early land plants. The extraordinary anatomical detail they preserve is a consequence of exceptional preservation, by silicification, in the hot-springs environment they inhabited. However, this has prompted questions as to just how typical of early land plants the Rhynie chert plants really are. Some have suggested that they were highly adapted to the unusual hot-springs environment and are unrepresentative of ‘normal’ plants of the regional flora. New quantitative analysis of dispersed spore assemblages from the stratigraphical sequence of the Rhynie outlier, coupled with characterization of the *in situ* spores of the Rhynie chert plants, permits investigation of their palaeoecology and palaeophytogeography. It is shown that the Rhynie inland intermontane basin harboured a relatively diverse flora with only a small proportion of these plants actually inhabiting the hot-springs environment. However, the flora of the Rhynie basin differed from coeval lowland floodplain deposits on the same continent, as it was less diverse, lacked some important spore groups and contained some unique elements. At least some of the Rhynie plants (e.g. *Horneophyton lignieri*) existed outside the hot-springs environment, inhabiting the wider basin, and were indeed palaeogeographically widespread. They probably existed in the hot-springs environment because they were preadapted to this unstable and harsh setting.

This article is part of a discussion meeting issue ‘The Rhynie cherts: our earliest terrestrial ecosystem revisited’.

## 1. Introduction

The discovery of the Rhynie chert and the detailed monographing of its anatomically preserved plants [1–5] marked a turning point in research on the early land plant fossil record. Prior to this, research on early land plant fossils was more-or-less confined to that of Dawson on Lower Devonian coalified compression material from eastern Canada [6]. However, this work had been largely neglected by the scientific community [7]. The Rhynie chert was critical because the exquisite preservation via permineralization permitted three-dimensional interpretation of the cellular structure of the plants. This enabled a much better understanding of the anatomy and biology of the plants than that offered by coalified compressions that presented only a limited picture of their general morphology. To this day the Rhynie cherts provide by far the most useful material for analysing early land plants, and our knowledge of them is highly dependent on information gleaned from this source. However, despite the celebrations by contemporary botanists following their discovery (e.g. [8]), almost immediately elements of the scientific community began to question just how representative of early land plants they were (e.g. [9]). After all, they appeared to inhabit a peculiar hot-springs environment, which ultimately was responsible for their remarkable preservation. Is it possible that the Rhynie plants were of limited distribution and highly adapted to the unusual environment they inhabited, and consequently unrepresentative of the majority of early land plants that were more widespread and inhabited ‘normal’ environments beyond the hot-springs? In this paper, I will consider new quantitatively analysed evidence from integrated analysis of *in situ* spores and dispersed spore assemblages pertinent to the ecology and palaeophytogeography of the Rhynie chert plants.

## 2. Geology and environment of the Rhynie basin

For many years the Rhynie cherts were considered to be of Mid Devonian age. However, Richardson [10] recovered dispersed spore assemblages from surface exposure which, despite being poorly preserved and highly coalified, indicated an Early Devonian age. More recently dispersed spore assemblages have been recovered from borehole material from throughout the stratigraphical sequence of the Rhynie outlier [11]. This demonstrated that the entire sequence belongs to the *polygonalis*–*emsiensis* Spore Assemblage Biozone indicating a Pragian to ?earliest Emsian age.

The Rhynie deposits accumulated on the southeast margin of Euramerica (Old Red Sandstone continent) at approximately 25°S. They represent deposits that accumulated in a small half-graben located within the rapidly eroding remnants of the Caledonian Orogeny (i.e. it was essentially an intermontane basin). Examination of the stratigraphy and sedimentology of the Rhynie deposits indicates that an axial river flowed northward through the basin and regular flooding distributed overbank deposits across the drainage basin [12–16]. To the south the Caledonian Mountains gave way to lowland floodplain deposits of Southern Britain that drained southward into the shallow marine deposits on the southeast shores of Euramerica [17]. The former are represented by the Lower Old Red Sandstone deposits of the Anglo-Welsh Basin while the latter are represented by the shallow marine deposits of the Ardennes-Rhenish region.

The climate is believed to have been arid to semi-arid (probably with seasonal rainfall) [17,18]. The nature of the atmosphere at this time is controversial but models such as GEOCARBSULF suggest O_2_ levels similar (26%) and CO_2_ levels much higher (3100 ppm) than at present (e.g. [19]).

The Rhynie half graben subsided rapidly preserving more than 200 m of sediments (that also incorporated some extrusive volcanics, including a lava flow). The northward flowing axial river that flowed through the basin deposited fluvial sediments and overbank flood deposits. Most of the sediments consist of fluvial and lacustrine sands, silts and muds. At the fault-bounded margin of the half-graben, there was significant hydrothermal activity and the Rhynie cherts are the surface manifestation of this. Plants appear to have grown on both sand and sinter surfaces. The sand was deposited by regular flooding of the river system and the sinter was deposited by the hot-springs. Plants rooted in sands and sinters appear to have colonized these surfaces between episodes of flooding and pulses of sinter deposition, respectively. However, it was the periodic inundation of silica-rich waters from the hot-springs that preserved the plants in sinter. This subsequently was transformed into solid cherts during burial and as a consequence of percolation of hydrothermal fluids from below.

There is some debate regarding the nature of the hydrothermal waters emanating from the vents and geysers (temperature, pH, redox potential, salinity, nature of dissolved minerals). Rice *et al*. reported on detailed geochemical analysis of the Rhynie cherts and concluded that they were deposited from a low-salinity fluid (0.1–0.3 wt% NaCl) of probable meteoric origin at temperatures of between 90 and 100°C [15]. However, it was later suggested that the original sinter was deposited at lower temperatures before being buried and converted into solid chert at these higher temperatures [14]. Analysis of fluid inclusions in Rhynie chert suggests they were deposited in temperatures of less than 40–50° and were often saline (10 ppt including *ca* 1 wt% NaCl) [20]. Indeed, similarities between certain features in the cool (less than 30°C) distal areas of sinter outwash aprons at modern Yellowstone National Park have been noted [21]. Interestingly, no fish fossils have yet been reported from the Rhynie cherts Unit suggesting that the aquatic environments were too hostile for fish survival.

With all of the above in mind, it is important to recall that hot-springs environments tend to be highly variable. The circuitous plumbing switches rapidly and there is a complex association with local river/pond/ground water. Thus the physical and chemical characteristics of water flowing from vents/geysers and that in local ponds vary dramatically and rapidly both spatially and temporally. In modern hot-springs systems, this results in a highly complicated association between hot-springs environments and the vegetation they harbour (summarized in [22]).

It seems likely that the water table was close to the surface with frequent ponding [21,23]. There is no evidence that the plants were totally submerged (i.e. aquatic). Indeed, Kidston & Lang ruled out this possibility for some of the plants based on the presence of stomata at the base of upright axes. However, it does seem likely that some stands of plants were from time-to-time partially submerged. At least some of the plants could survive this based on the fact that they have stromatolitic growths around their bases from cyanobacterial mats inhabiting the ponds, although it has recently suggested that the stromatolite possibly supported the plants post-mortem [24]. The presence of pyrite in the sediments suggests that subsurface conditions were water-logged and reducing.

At Rhynie, we appear to have active geysers and vents outflowing across sinter systems into the alluvial floodplain (although geysers/vents and sinters are actually underrepresented in the cherts they must have been present). Streams and ponds of both hot-springs waters and normal meteoric/fluvial waters would have occurred across the sinters and merging into the freshwater ponds and streams of the floodplain. Frequent flooding from the axial river deposited sediments across the sinters and it would appear that rapid subsidence of the basin meant that fluvial/lacustrine sedimentation often outstripped sinter deposition. In the main Rhynie cherts Unit, there are over 50 cherts but more than 85% of the sediment thickness constitutes fluvial/lacustrine deposits (e.g. [25]). Powell *et al*. estimate that approximately 40% of the ground cover in the active hot-springs area was by plants [25].

Clearly, the Rhynie chert plants inhabited this highly unstable environment and it appears that there was never enough time for a climax community to develop. Indeed coeval plants present in the drainage basin, as evidenced by the diverse dispersed spore assemblages, are not present in the cherts. Thus the Rhynie chert plants are likely to have been capable of rapidly colonized new sediment/sinter surfaces (via spore dispersal) and quickly establishing clonal colonies. These were then wiped out, or at least partially destroyed, by the next flood or inundation by hot-springs waters. In all likelihood, the plants were resilient to the unstable and often harsh environment and may have been tolerant of partial submersion, heat and unusual water chemistry (with respect to pH, salinity, heavy metals, etc.). But what is interesting is whether these plants were adapted and limited to existing in a hot-springs environment, whether they inhabited the mesic environments and were impinged on by the hot-springs system as it naturally migrated, or whether they were a part of the regional biota (be that the flora of the basin or also beyond in the mountains). Sadly, we have no plants from the regional biota preserved (although there is some evidence from coeval strata from elsewhere). In the succeeding section, I will discuss previous interpretations of the physiology, palaeoecology and palaeophytogeography of the Rhynie chert plants.

## 3. A brief history of the debate regarding the nature of the Rhynie chert plants

Early interpretations of the Rhynie cherts invoked deposition from fumaroles, geysers and hot springs (e.g. [26]), and Kidston & Lang [1–5] suggested that the plants were preserved by silicification of peat by siliceous waters emanating from such hot-springs. They noted that *in situ* growth indicated that the peat formed by the growth of plants ‘on the spot’ but that there was periodic inundation by water that was responsible for the deposition of sandy layers within the peat sequence. Kidston & Lang made further interpretations based on anatomical features of the plants [5]. They noted that *Rhynia* and *Asteroxylon* have stomata from the base of their upright stems and probably were not therefore truly aquatic, although they conceded that the soil could have been swampy or saturated. They further noted that *Horneophyton* may have grown in shallow water but found no evidence to support this.

Almost immediately after the Rhynie chert plants were first described a number of prominent palaeobotanists suggested that these remarkably preserved plants might be highly adapted plants peculiar to the strange ecological conditions they inhabited and consequently not a good model for early land plants. Scott [27, p. 391] noted that ‘It is, however, not impossible that the modest peat plants of the Rhynie Flora might have already undergone some reduction…’ [27]. Subsequently, Scott [28, p. 184] warned ‘We must bear in mind, however, that we are dealing with a limited and very special Flora, the vegetation of an old peat-bed, growing under conditions not by any means advantageous, and therefore not necessarily to be taken as typical of the plant-life of the period’ [28].

Following a period of quiescence in Rhynie research Tasch re-examined the data from the original trenches into the Rhynie chert [29]. He concluded that the cherts formed when ponds intermittently charged with hot silicic waters flooded the land surface. He estimated that the entire 3.7 m sequence accumulated in 1000 ± 200 years with the basal peat horizon accumulating in 150 ± 50 years.

An important advance was Richardson's realization that the Rhynie cherts were Early rather than Mid Devonian in age [10]. This older age meant that the Rhynie chert plants were not so simple compared with coeval plants as had previously been considered. In fact they were much more in keeping with coeval floras from Gaspé, the Anglo-Welsh Basin, the Ardenne-Rhenish region, etc.

**Figure 1.**
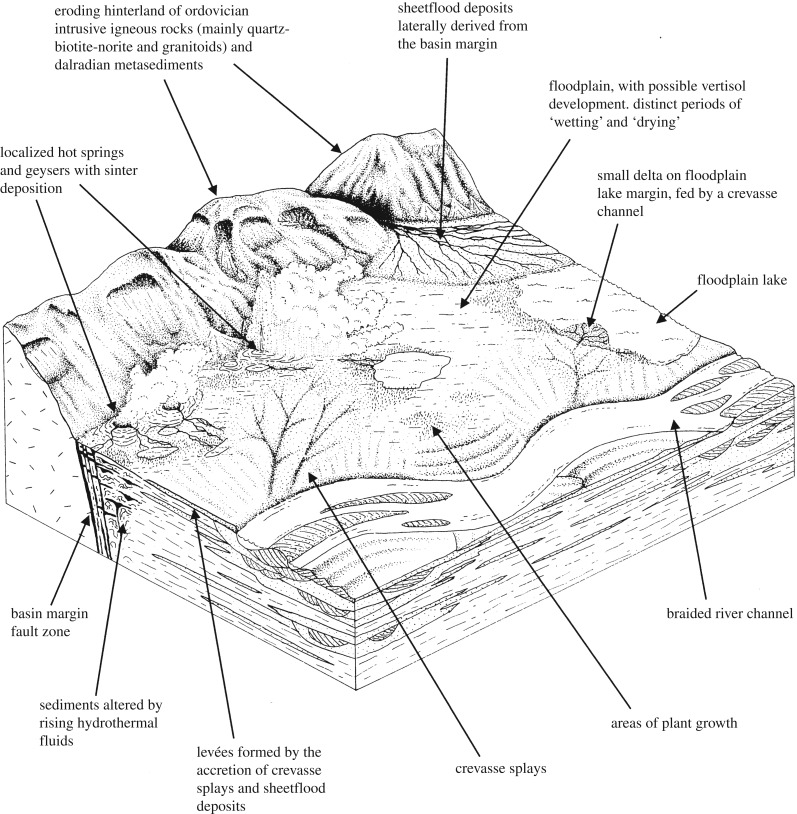
Reconstruction of the Rhynie basin depositional setting and environments. From Fayers & Trewin [30]. Reproduced with permission of The Royal Society of Edinburgh from *Transactions of the Royal Society of Edinburgh: Earth Sciences* volume 94(4) (2004, for 2003), pp. 325–339.

In 1988, the first boreholes were drilled into the Rhynie sequence and with this came a revolution in understanding of the geology of the Rhynie Outlier and the nature of the cherts [12–16,25]. The new material enabled clarification of the geological structure and stratigraphical sequence developed in the Rhynie outlier. Thus the Rhynie cherts could be considered within a secure geological context for the first time. It soon became evident that at least 50 chert horizons were present interspersed within *ca* 35 m of sediments of the Rhynie cherts Unit. A further chert-bearing horizon was recognized as the Windyfield chert [30]. The cherts were of limited lateral extent and some were stacked and they represented less than 15% of the thickness of the stratigraphical unit [25]. Further boreholes were drilled between 1997 and 2000 enabling even better understanding of the Rhynie Outlier [21,31]. A general consensus was reached that the Rhynie cherts formed in a wetland where there was active sediment deposition from overbank flooding of an axial river and active sinter deposition from the activity of hot-springs. Trewin interpreted the Rhynie chert plants as growing close to pools on a fluvial floodplain either on sandy substrates or sinter surfaces. The silica in which they are preserved was sourced from a hot-spring system with the cherts representing sinters of surficial origin [13] (figure 1).

**Figure 2.**
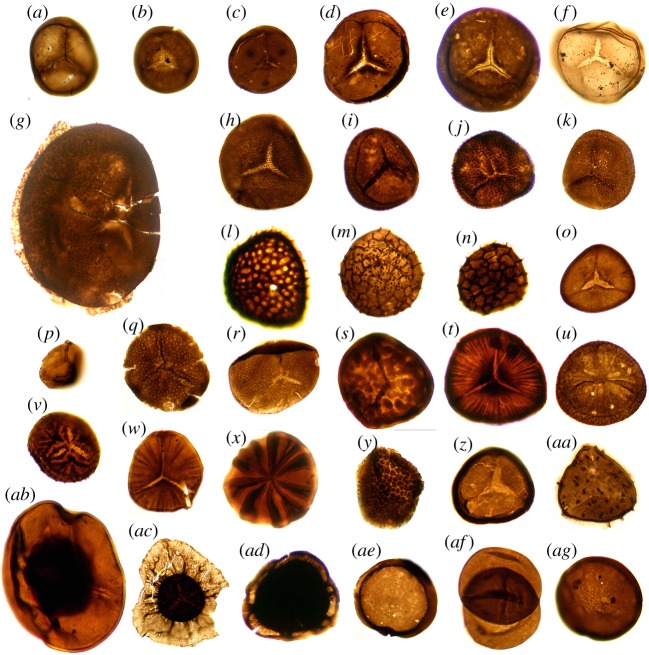
Light microscope images of the common dispersed spore taxa from the Rhynie assemblage (for more details of the spores see [11]). All specimens at magnification ×300. (*a*) *Retusotriletes* spp. (*b*) cf. *Retusotriletes fayersii* Wellman 2006 (*c*) *Retusotriletes maculatus*? McGregor & Camfield 1976 (*d*) *Retusotriletes* cf. *triangulatus* (Streel) Streel 1967 (*e*) *Retusotriletes* sp. CW-A. Wellman 2006 (*f*) *Retusotriletes* sp. CW-B Wellman 2006 (*g*) *Apiculiretusispora brandtii* Streel 1964 (*h*) *Apiculiretusispora plicata* (Allen) Streel 1964 (*i*) *Apiculiretusispora* sp. CW-A Wellman 2006 (*j*) *Apiculiretusispora* sp. CW-B Wellman 2006 (*k*) *Apiculiretusispora* sp. CW-C Wellman 2006 (*l*) *Dictyotriletes*? *hagenii* Wellman 2006 (*m*) *Dictyotriletes kerpii* Wellman 2006 (*n*) *Dictyotriletes subgranifer* McGregor 1973 (*o*) *Ambitisporites* sp. CW-A Wellman 2006 (*p*) *Aneurospora* sp. CW-A Wellman 2006 (*q*) *Brochotriletes* sp. CW-A Wellman 2006 (*r*) *Brochotriletes rarus* Arkhangelskaya 1978 (*s*) *Clivosispora verrucata* McGregor 1973 (*t*) *Emphanisporites edwardsiae* Wellman 2006 (*u*) *Emphanisporites* cf. *decoratus* Allen 1965 (*v*) *Emphanisporites zavallatus* Richardson, Streel, Hassan & Steemans 1982 (*w*) *Emphanisporites rotatus* McGregor 1961 (*x*) *Emphanisporites robustus* McGregor 1961 (*y*) *Verrucosisporites polygonalis* Lanninger 1968 (*z*) *Archaeozonotriletes chulus* (Cramer) Richardson & Lister 1969 (*aa*) *Stalicospora trewinii* Wellman 2006 (*ab*) *Leiozosterospora andersonii* Wellman 2006 (*ac*) *Camptozonotriletes*? *caperatus* McGregor, 1973 (*ad*) *Camptozonotriletes*? sp. CW-A Wellman 2006 (*ae*) *Laevolancis divellomedium* (Cramer) Burgess & Richardson 1991 (*af*) *Dyadospora murusdensa* Strother & Traverse, 1979, emend. Burgess & Richardson 1991 (i.e. a dyad of *Laevolancis divellomedium* (Cramer) Burgess & Richardson 1991) (*ag*) *Laevolancis* sp. CW-A Wellman 2006.

During this new phase of research, plant occurrences were examined in individual cherts present in trenches and boreholes. The former offered limited vertical exposure but reasonable lateral exposure. The latter offered excellent vertical exposure but very limited (cm scale) lateral exposure. Thus Powell *et al*. were able to estimate that plant cover was around 40% and ascertain which plants colonized what substrates, co-occurrence of plant types, etc. [25].

Drilling of the boreholes also permitted detailed palynological analysis of the stratigraphical sequence present in the Rhynie Outlier. Wellman described dispersed spore assemblages from throughout the sequence and was able to interpret the nature of the regional biota [11] and also integrated analysis of *in situ* spores from the Rhynie chert plants and dispersed spore assemblages from the Rhynie Outlier to interpret the ecology and palaeophytogeography of the Rhynie chert plants [32]. He concluded that at least some of the Rhynie chert plants (*Horneophyton*, *Aglaophyton*, *Rhynia*) were components of the regional flora and were preadapted to survive in the harsh environments of the hot-springs. This work is extended in this paper based on more refined spore occurrences/taxonomy and statistical analysis.

Numerous workers have analysed the exquisitely preserved anatomy of the Rhynie chert plants with a view to understanding their physiology and any evidence for particular adaptations for survival in the stressed hot-springs environment (e.g. [5,33–36]). While this has provided some evidence for high water use efficiency, it can provide little evidence for tolerance to heavy metals, alkalinity, salt, silica, etc. Modelling has also been attempted and provides some evidence regarding water transport and gas exchange [37,38]. These findings led Channing & Edwards to suggest that either water was not consistently available in the substrate or it was present but not readily available [22].

Other workers have studied modern analogues of the Rhynie hot-springs system to gain an insight into the ecology of the plants. This can provide information on: (i) the type and distribution of modern vegetation in and around hot-springs; (ii) the ecophysiology of the plants involved; (iii) how the plants become silicified. Particular attention has recently been paid to the Yellowstone National Park (USA) hot-springs system [22,23,39,40] and a number of important observations made. However, when making comparisons between Rhynie and Yellowstone one should be wary that there are environmental differences: Yellowstone is essentially a drying/oxidizing environment with a low water table, whereas Rhynie was probably reducing/saturated (based on the presence of pyrite and reduced sediments at Rhynie); Yellowstone has a harsh continental climate with extremes of heat/cold, whereas the Rhynie climate was probably more moderate; large, trampling, herbivorous, dung-producing animals are present at Yellowstone; diatoms influence the Yellowstone siliceous system, etc. [22,23,39,40]. Despite these differences a number of important observations are pertinent. Channing & Edwards note that wetland conditions are a requirement for preservation of the Rhynie-type and that most plants silicified in hot-springs environments are flooding tolerant and inhabit areas with high water table and saturated substrates [22]. Furthermore, they note that the commonest plants in the Yellowstone geothermal wetlands are salt-marsh colonizers and tolerant of high salinity. Based on comparisons between Yellowstone and Rhynie they concluded that the Rhynie chert plants were at least flooding tolerant and could cope with fluids that for many dryland plants would be toxic (i.e. were physiologically specialized to withstand chemical stress). They concluded that they were probably not endemics but were not common elements of the mesophytic vegetation and most probably were confined to water stressed environments.

Despite recent claims [41], it is my opinion that there is no definitive macrofossil evidence for the presence of Rhynie chert plant taxa from beyond the Rhynie chert hot-springs environment. This is most likely because (i) the Rhynie chert plants were largely of parenchymatous construction and therefore of low fossilization potential; (ii) only a very limited representation of the regional flora is ever preserved (compare the number of distinctive spore taxa known compared with the known plant megafossils) and this almost certainly reflects fossilization bias towards plants that were of sturdy construction and inhabited areas where they were likely to be incorporated into sediments; (iii) it is very difficult to compare plant fossils preserved in different ways (i.e. permineralization versus coalified compression). That said, it is possible that the exceptionally preserved charcoalified plant described from the Early Devonian (Lochkovian) of the Anglo-Welsh Basin [42] represents a species of the taxon *Horneophyton* based on identification of its distinctive *in situ* spores [43].

From the brief review above, it is evident that understanding of the Rhynie chert hot-springs environment has increased dramatically over recent years based on new evidence from geology (analysis of the boreholes) and comparisons with modern environment analogues (e.g. Yellowstone). However, despite our great understanding of the morphology, anatomy and life cycle of the Rhynie chert plants, interpretation of their ecophysiology remains conjectural.

## 4. Analysis of *in situ* and dispersed spores

### (a) Material and methods

Dispersed spore assemblages from the Rhynie outlier have been described in a study of 106 productive samples distributed throughout a large part of the stratigraphical sequence [11]. Relative abundance counts were undertaken on 70 of the best-preserved samples. In this paper, these dispersed spore assemblages are compared with those from coeval deposits of the Old Red Sandstone continent (Euramerica) from: (i) the Cosheston Group of the lowland floodplain deposits of the Anglo-Welsh Basin (analysing 18 samples) [44,45]; (ii) mixed marine/non-marine floodplain deposits of Gaspé, eastern Canada (analysing 28 samples) [46,47]; (iii) nearshore marine deposits of the Ardenne-Rhenish region (analysing 75 samples) [48]. A list of spore taxa reported from each of these regions is presented in electronic supplementary material, table S1 (synonymies provided by the author). In this compilation: (i) taxa represented by single occurrences or very low numbers are highlighted or omitted completely; (ii) taxa of simple laevigate spores are omitted, unless they have highly distinctive features and are easily identified, and are listed simply as *Retusotriletes* spp. (laevigate retusoid), *Ambitisporites* spp. (laevigate crassitate) or *Archaeozonotriletes* spp. (laevigate patinate). Such simple spores are very difficult to assign to species and different workers tend to treat them very differently. Consequently, they were omitted in order that they did not skew the analyses.

The species lists for all four regions were analysed with respect to how similar they are using two metrics: (i) the coefficient of similarity (CS) *sensu* Clark & Harteberg [49]; (ii) the Jaccard Index (Jaccard Similarity Coefficient) (JI). The former is widely used in bioprovincialism evaluation of extant biotas and has been applied in numerous Palaeozoic palynological studies (e.g. [50]). It can be expressed as CS(*a,b*) *=* 2|*a*Π*b*|/|*a* + *b*| (where *a* and *b* are the total number of species in assemblage *a* and *b*, respectively). The latter is widely used in ecological studies of extant biotas and has also been applied in Palaeozoic palynological studies (e.g. [51]). It can be express as JI(*a*,*b*) = |*a*Π*b*|/(|*a*|+|*b*| – |*a*Π*b*|) (where *a* and *b* are the total number of species in assemblage *a* and *b*, respectively) . Regarding CS, if CS < 0.2 the similarity between the two assemblages is considered to be low, if the CS is between 0.2 and 0.55 the similarity is considered as moderate to high, and if CS > 0.55 the similarity is considered to be very high [49].

*In situ* spores have been identified in all of the Rhynie chert plants (table 1). This enables consideration of the distribution of the Rhynie chert plants' spores within the dispersed spore assemblages from Rhynie and the other three localities on the Old Red Sandstone Continent.

**Table 1.** Details of the *in situ* spores of the Rhynie chert plants. 1 = Highly distinctive spore very unlikely to be produced by other plants due to convergence; 2 = Moderately distinctive spore possibly produced by other plants due to convergence; 3 = Very common and indistinct spore type produced by numerous plant type due to convergence.

plant (affinities)	spore	occurrence	reference
*Aglaophyton majus* (pro-tracheophyte)	*Retusotriletes* sp. CW-A	2	[59]
*Horneophyton lignieri* (pro-tracheophyte)	*Emphanisporites* cf. *decoratus*	1	[60]
*Rhynia gwynne-vaughanii* (rhyniophyte)	*Apiculiretusispora plicata*	2	[11,32]
*Trichopherophyton teuchansii* (zosterophyll)	*Retusotriletes* cf. *triangulatus*	3	[11,32]
*Ventarura lyonii* (zosterophyll)	?*Retusotriletes* cf. *triangulatus*	3	[11,32]
*Nothia aphylla* (?)	*Retusotriletes* sp.	3	[11,32]
*Asteroxylon mackiei* (Drepanophycaceae)	*Retusotriletes* cf. *triangulatus*	3	[61]

### (b) Results

Wellman recognized that the dispersed spore assemblages recovered from throughout the Rhynie sequence were all very similar and all essentially represented the same assemblage [11]. He equated this with the *polygonalis*–*emsiensis* Spore Assemblage Biozone (PE SAB) of Richardson & McGregor [52] and the PoW Oppel Zone (PoW OZ), potentially Su Interval Zone (Su IZ), of Streel *et al*. (1987) [53]. This suggests an early (but not earliest) Pragian to ?earliest Emsian age (potentially a late Pragian to ?earliest Emsian age based on recognition of the Su IZ).

The Rhynie dispersed spore assemblages contain 31 taxa that are common and easily recognized. However, a further 42 taxa were illustrated that were represented by very few occurrences (predominantly singletons). Comparisons with the spore assemblages from elsewhere on the Old Red Sandstone continent are provided in electronic supplementary material, table S1, with table 2 presenting data on the number of samples examined and the number of taxa reported. These data show that: (i) the Rhynie dispersed spore assemblages are less diverse than those of the Cosheston Group, Gaspé and the Ardenne-Rhenish region; (ii) spore morphotypes under-represented at Rhynie include *Dibolisporites* spp. and ornamented patinate spores (*Chelinospora* spp. and *Cymbosporites* spp.); (iii) three taxa appear to be endemic to Rhynie: *Dictyotriletes kerpii*, *Brochotriletes* sp. CW-A and *Camptozonotriletes*? sp. CW-A. The results of statistical analyses of similarity are presented in table 3.

**Table 2.** Details of numbers of samples examined and distinctive spore taxa identified in spore assemblages from the different sequences.

locality	samples	common distinctive taxa
Rhynie, Scotland	70	31
southwest Wales	18	46
Gaspé, Canada	28	38
Ardenne-Rhenish region	75	88

**Table 3.** Comparison of similarity between spore assemblages from the different sequences. The number in italics is the number of shared taxa. The first number in parenthesis is the coefficient of similarity (CS). The second number in parenthesis is the Jaccard Index (JI).

	Rhynie, Scotland	southwest Wales	Gaspé, Canada	Ardenne-Rhenish region
Rhynie, Scotland		*21* (0.55/37.5)	*14* (0.41/25.9)	*15* (0.25/14.4)
southwest Wales	*21* (0.55/37.5)		*19* (0.45/29.7)	*29* (0.43/27.6)
Gaspé, Canada	*14* (0.41/25.9)	*19* (0.45/29.7)		*23* (0.37/22.5)
Ardenne-Rhenish region	*15* (0.25/14.4)	*29* (0.43/27.6)	*23* (0.37/22.5)	

The spores of the Rhynie cherts plants can be recognized among the dispersed spore assemblages from the Rhynie outlier with varying degrees of confidence (table 1). *Horneophyton* has extremely distinctive spores (*Emphanisporites* cf. *decoratus*) and can be recognized with confidence within the Rhynie assemblages and within other assemblages described from elsewhere. *Rhynia* has reasonably distinctive spores (*Apiculiretusispora brandtii*) that can be recognized with confidence within the Rhynie assemblages. However, identification within other more diverse assemblages from elsewhere in the world is considered unsafe because similar spores have been reported *in situ* from a number of plant types [54–57] and it seems likely that this spore morphology has evolved numerous times due to convergence. It appears to be particularly common among the trimerophytes [58]. Similarly the spores of *Aglaophyton* (*Retusotriletes* sp. CW-A) can be recognized within the Rhynie assemblages where it is common and well circumscribed, but not with confidence in more diverse assemblages from elsewhere in the world where often a number of similar spore morphotypes occur. Spores of all of the other Rhynie plants belong to *Retusotriletes* spp. This taxon is not very distinctive and has been reported *in situ* from many plant types (particularly from zosterophylls) [54–57]. Thus it is unsafe to identify its parent plant within the Rhynie assemblages and certainly in other assemblages from elsewhere.

### (c) Interpretation

This diversity of the Rhynie dispersed spore assemblage is lower than all of the other sequences, despite the fact that the Rhynie sequence was one of the most exhaustively sampled with 70 productive samples logged. This suggests that the Rhynie flora was somewhat depauperate compared to the others. The most likely explanation is that the Rhynie flora represents one existing in an inland intermontane basin where the environment was more stressed than that in the lowland floodplain deposits represented by the deposits from southwest Wales and Gaspé. The very high taxon diversity reported from the Ardennes-Rhenish region probably reflects the fact that these assemblages are from nearshore marine deposits into which numerous rivers drained bringing elements of the floras from throughout the entire drainage basin (i.e. representing the inland intermontane basin and floodplain deposits). Further evidence that the Rhynie spore assemblage represents an inland intermontane basin flora is the presence of taxa unique to Rhynie, such as *Dictyotriletes kerpii*, *Brochotriletes* sp. CW-A and *Camptozonotriletes*? sp. CW-A, that may represent taxa endemic to the inland uplands (electronic supplementary material, table S1).

The spore assemblages from the different sequences may be compared in terms of similarity by analysing the number of shared taxa using the Coefficient of Similarity and Jaccard Index (results presented in table 3). It is clear that the Rhynie assemblage is most like those from the lowland floodplain deposits of southwest Wales and Gaspé and least like those from the Ardennes-Rhenish region. Regarding the floodplain deposits southwest Wales is more similar than Gaspé, probably because the former is geographically closer, with Gaspé further south at higher latitude. Compared to the floodplain deposits it is noticeable that Rhynie has many fewer patinate spores (particularly ornamented forms belonging to *Cymbosporites* and *Chelinospora*), *Dibolisporites* is less abundant, and certain distinctive and common taxa are absent from Rhynie (e.g. *Breconisporites breconensis* and *Streelispora newportensis*).

It is evident that the seven plants preserved in the Rhynie cherts represent only a subset of the plants that flourished throughout the Rhynie drainage basin. Presumably the majority of the flora was excluded from the hot-springs environment because they were unable to grow in the unstable and harsh setting. Nonetheless, in the cases where we can confidently recognize spore producers within the Rhynie sequence, it is clear that spores from *Horneophyton*, *Rhynia* and *Aglaophyton* are represented in all the spore assemblages examined. These come from below the chert bearing unit, within and between the chert bearing horizons, and above the chert-bearing unit. This strongly suggests that these three plants were a component of the flora of the Rhynie drainage basin throughout its existence and not just when the hot-springs were active. These observations suggest that these three plants were not specialists adapted to and confined to hot-springs environments, but existed throughout the drainage basin as a common element of the flora [32]. Furthermore, they imply that these three plants were preadapted to exist in the hot-springs environment [32]. Most probably they were stress-tolerant ruderals that could: (i) rapidly colonize newly created unstable environments and grow quickly to maturity, before the environment was once again disrupted by inundation of either flood waters, bringing sand from the river system or sinter depositing hot-springs waters; (ii) tolerate water stress and perhaps also extremes of temperatures, pH, salinity and heavy metals concentration.

Of course it may be that these three plants inhabited unstable and challenging environments in other parts of the drainage basin (and beyond) where they were able to tolerate periodic submergence, elevated salinity, etc. In fact such environments may have been frequent in the inland uplands. For example, Lower Old Red Sandstone deposits further north in the Northern Highlands of Scotland include hostile playa mudflat facies and interlaminated bituminous dolomites with evidence for the precipitation of gypsum [18]. The Rhynie plants may also have thrived in these and other harsh environments.

Unfortunately, due to the indistinct morphology of their spores, similar analysis cannot be conducted on the other plants of the Rhynie chert (*Asteroxylon*, *Nothia*, *Trichopherophyton*, *Ventarura*). It could be that these plants were indeed highly adapted specialists that flourished in hot-springs environments.

## 5. Conclusion

— Analysis of dispersed spore assemblages suggests that the inland intermontane Rhynie basin harboured a diverse flora, of which the seven plant taxa preserved in the Rhynie chert were only a subset, with the remaining plants excluded from the harsh hot-springs environment.
— The Rhynie basin flora was distinct from coeval lowland floodplain floras in that is was less diverse, lacked some distinctive spore/plant groups, but also contained some unique elements.
— One of the Rhynie chert plants, *Horneophyton lignieri*, appears to have been a conspicuous element of the Rhynie basin flora and was palaeogeographically widespread over Euramerica.
— Two of the Rhynie chert plants, *Rhynia gywnne-vaughanii* and *Aglaophyton majus*, were also conspicuous element of the Rhynie basin flora, but cannot be identified with confidence beyond the Rhynie basin and it is unclear how palaeogeographically widespread they were.
— It is not possible to use spores to identify the distribution of the other four Rhynie chert plants.
— *H. lignieri*, *R. gywnne-vaughanii* and *A. majus* were common elements of the regional biota (and possibly beyond) but were preadapted to survive in the harsh hot-springs environment. This is most likely because they were stress-tolerant ruderals capable of colonizing unstable environments, able to survive periodic submergence, and tolerant of water stress and perhaps also extremes of temperatures, pH, salinity and heavy metals concentration. They may have inhabited playa lake and other hostile environments that were common in the inland uplands.
— The other Rhynie chert plants may also have been restricted to stressed hot-springs environments but evidence from spores has no bearing on this debate.

## Supplementary Material

Supplemetary Table
